# 

**Published:** 1884-05

**Authors:** 


MAY, 1884.

THIRTY-FIRST YEAR.

### HALL'S JOURNAL OF HEALTH

Guaranteed Circulation 200,000 Copies in 12 Months.

*E. H. GIBBS, A.M., M.D., Editor,*

No. 21 CLINTON PLACE EIGHTH STREET NEW YORK.

TERMS: $1 a Year. Single number, 10 cts.

